# Characterization and Prediction of the Clinical Outcome of Intense Pulsed Light-Based Treatment in Dry Eye Associated to Meibomian Gland Dysfunction

**DOI:** 10.3390/jcm10163573

**Published:** 2021-08-13

**Authors:** María T. Iradier, María Ángeles del Buey, Cristina Peris-Martínez, Priscilla Cedano, David P. Piñero

**Affiliations:** 1 Iradier Eye Clinic, 28035 Madrid, Spain; clinicairadier@gmail.com (M.T.I.); priscilacedano@hotmail.com (P.C.); 2 Department of Ophthalmology, Hospital Clínico Universitario “Lozano Blesa”, 50009 Zaragoza, Spain; madelbuey@gmail.com; 3 Cornea and External Diseases Unit, FISABIO-Oftalmología Médica (FOM), 46015 Valencia, Spain; peris_crimar@gva.es; 4 Clínica Oftalmológica Aviñó & Peris, 46001 Valencia, Spain; 5 Department of Optics, Pharmacology and Anatomy, University of Alicante, 03690 San Vicente del Rapeig, Spain

**Keywords:** intense pulsed light, dry eye, meibomian gland dysfunction, tear osmolarity, OSDI

## Abstract

This non-comparative prospective case series was conducted to characterize the clinical impact of intense pulsed light (IPL)-based treatment in dry eyes associated to Meibomian gland dysfunction (MGD), defining the predicting factors for a successful outcome with this therapy in a large case series. A total of 390 eyes (195 patients, range: 23–93 years) received four sessions of Optima IPL system (Lumenis, Yokneam, Israel). Significant changes were observed in tear film osmolarity in both eyes (*p* < 0.001) and in meibum quality (*p* < 0.001), with more eyes showing clear or yellow secretions after therapy. Mean change in the ocular surface disease index (OSDI) was −8.61, ranging from −27.00 to 11.00. This change was significantly correlated with the baseline value of OSDI (r = −0.489, *p* < 0.001). The change in osmolarity correlated significantly with the baseline osmolarity in both eyes (right r = −0.636, left r = −0.620, *p* < 0.001). A linear predicting model of the change in OSDI with therapy was obtained: change OSDI = 10.99 − 0.35 × OSDI − 1.03 × NIBUT_RE-LE_ (mean non-invasive break up time of right and left eye) − 2.03 × Meibum quality grade (*p* = 0.001; R^2^: 0.325). In conclusion, the improvement in symptomatology achieved with an IPL-based therapy can be predicted at baseline using a linear model considering the level of MGD and the magnitude of OSDI and NIBUT (non-invasive break-up time).

## 1. Introduction

Intense pulsed light (IPL) therapy has been used since many years ago in dermatology for the treatment of a great variety of conditions, including pigmented lesions, benign cavernous hemangiomas, telangectasias or facial rosacea [1]. The analysis of the outcomes of IPL treatment in this last condition led to the discovery of the potential of this therapeutic option for the treatment of dry eye due to Meibomian gland dysfunction (MGD) [2,3,4]. Since then, a great variety of studies have been conducted to demonstrate the efficacy of IPL for the treatment of dry eye associated to MGD without [5,6,7,8,9,10,11,12,13,14,15,16] and with the combined meibomian gland expressibility (MGX) [17,18,19,20,21,22]. The American Academy of Ophthalmology have recently published a report on the efficacy of IPL treatment for MGD, concluding that the existing body of the scientific literature demonstrates improvements in the signs of symptoms of this condition, although methodological limitations and potential conflicts of interest were present in some studies [23]. Likewise, other ocular therapeutic uses of IPL have been investigated in conditions such as the treatment of refractory aqueous-deficient dry eye accompanied by mild MGD [24], post-laser in situ keratomileusis (LASIK) dry eye [25,26], Demodex [27] or MGD in skin types III/IV [28].

From a technical perspective, the IPL therapy is based on the delivery of intense pulses of noncoherent light with wavelengths from 500 to 1200 nm that are normally applied to the lower lid and temporal lid margin. This process is repeated in 2–4 sessions [29]. This irradiation of filtered polychromatic broad-bandwidth wavelengths with varying pulse durations generates various effects that contribute to its therapeutic properties: Meibomian glands warming that facilitates the expressibility and release of the meibum inside, improvement of the function of Meibomian glands, reduction of tear osmolarity, reduction of proinflammatory mediators contributing to dry eye, improvement of the cellular functions including fibroblasts regeneration, collagen synthesis, motility in immunoregulatory cells and photobiomodulation [15,30,31]. All these effects have a significant impact on different clinical measures. The aim of the current study was to characterize in a large series of cases the clinical impact of IPL-based treatment in dry eyes associated to MGD, defining the predicting factors for a successful outcome with this therapeutic approach. To our knowledge, this is the largest series of cases reported to this date treated with an IPL-based protocol.

## 2. Materials and Methods

### 2.1. Patients

This non-comparative prospective case series study enrolled a total of 202 patients ranging in age from 23 to 93 years old and with the diagnosis of dry eye associated to meibomian gland dysfunction, being good candidates for IPL treatment. A strict protocol of selection and treatment was followed in all cases involved in this research. Patient recruitment was conducted between December 2019 and November 2020. The study was conducted at Iradier Eye Clinic (Madrid) following the tenets of the Declaration of Helsinki. All patients were informed about the nature of the study and provided written consent before being included in the trial. 

Inclusion criteria were as follows: diagnosis of evaporative or mixed dry eyes with MGD associated, meibomian gland loss grading 2 to 4 in lower right and left eyelids according to the grading scale define by Heiko Pult (degree 0 = no partial glands; 1 ≤ 25% partial glands; 3 = 25–75% partial glands; 3 ≥ 75% partial glands) [32], acceptance and signature of the informed consent, satisfaction of the inclusion and manifest intention of following the protocol and attending to all study visits. The diagnosis of evaporative and mixed dry eye was performed according to the Tear Film and Ocular Surface Society (TFOS) Dry Eye Workshop (DEWS) II [33], considering the following criteria:

- Evaporative dry eye: OSDI (ocular surface disease index) ≥ 13, NIBUT (non-invasive break-up time) < 7 s, tear film osmolarity ≥ 308 mOsm/L, Schirmer I with topical anesthesia ≥ 7 mm in 5 min
- Mixed dry eye: OSDI ≥ 13, NIBUT < 7 s, tear film osmolarity ≥ 308 mOsm/L, Schirmer I with topical anesthesia < 7 mm in 5 min

Exclusion criteria for the study included use of medications producing increased photosensitivity, such as tetracyclines (oxytetracycline, chlortetracycline, demeclocycline, doxycycline or minocycline), sulfonamides (thiazides, sulfonylureas, and cyclamates), phenothiazine and derivatives (carbamazepine, chlorpromazine, promethazine and others), quinolones (ciprofloxacin, enoxacin, fleroxacin, pefloxacin, nalidixic acid and others), non-steroidal anti-inflammatory drugs (piroxicam, benoxaprofen, oxaprozin, tiaprofen, carprofen and others), antifungals (griseofulvin) and oral hypoglycemic agents (tolbutamide), pregnant women, patients being treated with Isotretinoin (patients were included at two months after stopping the use of this medication), presence of suspicious pigmentary lesion or possible skin cancer until it has been ruled out by biopsy by the dermatologist, patients who expect to be exposed to the sun for a long time during the treatment due to the risk of hypo or hyperpigmentation, active lesions in the area of the skin to be treated, solar lesions of less than a month of evolution in the area to be treated, and patients with unrealistic expectations.

### 2.2. Clinical Protocol

A complete ophthalmological examination was performed in all patients prior to the prescription and programming of the IPL treatment. This examination included measurement of uncorrected and corrected distance visual acuity, manifest refraction, meibography and measurement of the NIBUT with the Sirius system (CSO, Firenze, Italy), slit lamp biomicroscopy, Schirmer test with anesthesia (fluorescein + topic anaesthesia, 5 min), measurement of tear osmolarity (TearLab, TearLab Inc., Escondido, CA, USA), characterization of dry eye symptomatology using the validated questionnaire OSDI (Ocular Surface Disease Index) [34], predominant meibum quality in right and left lower eyelid of each patient (0, clear liquid; 1, yellow liquid; 2, granular; 3, solid) expressed using the Collins TM expressor forceps (OptiMed, Sydney, Australia) and fundus evaluation. The non-invasive meibography and measurement of NIBUT provided by the Sirius system has been previously demonstrated to be valid and precise [35,36]. This same examination was repeated one month after finishing the last IPL treatment session to confirm the efficacy of the therapy.

### 2.3. Treatment Sessions

All patients received 4 sessions of IPL treatment using the Optima IPL system (Lumenis, Yokneam, Israel) adjusted to the official optimized Lumenis setting (590-nm cutoff filter, triple pulses of 6.0 ms with an interval of 50 ms, and total fluence range of 11 to 14 J/cm^2^). Before initiating the treatment, each patient underwent a Fiztpatrick skin typing test [37] to determine the intensity of the pulsed light that would be administered. Specifically, lower powers (11/12) were used with darker skins whereas higher powers (13/14) were used for lighter skins. The large rectangular terminal was used for performing the IPL sessions.

At each treatment session, the patient was placed in a special chair to perform the treatment, allowing to maintain a comfortable position. The skin was cleaned of make-up residues or creams with micellar water. After this, both eyes of the patient were closed and sealed with special adhesive patches (IPL-Aid disposable eye shields, Honeywell Safety Products, Smithfield, VA, USA). If any type of hyperpigmentation was present (nevus, lentigines, etc.), it was covered by applying color with a special white pencil for such purpose. A layer of conductive gel for IPL was placed afterwards following the path of the skin on the lower eyelids from temple to temple, including the nose. A total of 5 impacts were then made in each region (right and left), with a total of 10 impacts in each application without overlapping then [2,20]. The terminal was always placed on the gel, without squeezing the skin. This procedure was repeated in a second pass, but after removing the previous gel used and replacing it by new gel. A total of 10 impacts were then applied again. The gel was then removed, and the skin was cleaned. Finally, anesthetic drops (0.4% oxybuprocaine hydrochloride) were applied to express the Meibomian glands with sterile forceps in the slit lamp, taking photos during the process to verify the evolution of the quality of the meibomian secretions. Preservative-free corticosteroid eye drops were applied. At least a period of two weeks was left between consecutive IPL treatment sessions. Follow-up after therapy ranged from 1 month to 7 months.

A different post-treatment pharmacological protocol was prescribed depending on the type of dry eye that was present. In evaporative dry eye, corticosteroid eye drops (Softacort, hydrocortisone 3.35 mg/mL, Thea Laboratories, Clermont-Ferrand, France) were prescribed to be applied every 8 h for 5 days, combined with application of local dry heat on eyelids for 5 min every day and use of artificial tears with lipid component every 3 h. In mixed dry eye, the following components were added to the treatment: cyclosporine 0.05% every 12 h, autologous serum 20% every 3 h or both simultaneously.

### 2.4. Data Analysis

The SPSS software package (SPSS Version 20.0; IBM Corporation, Armonk, NY, USA) was used to perform the analysis of data obtained in this study. Normality of data samples was first evaluated by means of the Kolmogorov-Smirnov test. The paired Student t and Wilcoxon tests were used for analyzing the statistical significance of the differences between pre-treatment and post-treatment visits when the data samples were normally and not normally distributed, respectively. The unpaired Student t and Mann-Whitney tests were used for assessing the statistical significance of the differences between independent groups when the data samples were normally and not normally distributed, respectively. The Chi-square test was used to compare percentage distributions. The Pearson or Spearman correlation coefficients were calculated to assess the degree of association between the change obtained in different variables and the magnitude of baseline parameters depending on if the data samples were or not normally distributed, respectively. 

Finally, a multiple linear regression analysis using the backward elimination technique was performed to define a linear model to predict the change in symptomatology (OSDI score) from baseline parameters. Durbin–Watson, tolerance and variation inflation factor indices were calculated to confirm whether there was collinearity between some parameters and to confirm whether there was not a significant correlation of errors in the regression model. Normality of unstandardized residuals was demonstrated using the Kolmogorov-Smirnov test, which is an elementary condition to accept the model. R^2^ and adjusted R^2^ were used to study the variance explained by the variables of the model.

## 3. Results

### 3.1. Demographics

A total of 390 eyes of 195 patients ranging in age from 23 to 93 years old were enrolled in the study (mean: 60.4, median: 63.0 years). The distribution of the sample in terms of gender was as follows: 54 males (27.7%) and 141 females (72.3%). A total of 20 patients (10.3%) underwent simultaneously a treatment with autologous serum and 29 patients (14.9%) had a simultaneous treatment with cyclosporine. Regarding ocular comorbidities, 26 patients (13.3%) had severe rosacea and 51 had mild rosacea (26.2%), 12 patients (6.2%) presented chalazion in at least one eye, four patients (2.1%) had Sjögren syndrome and 19 patients (9.7%) had undergone a previous laser in situ keratomileusis (LASIK) surgery. 

### 3.2. Changes in Schirmer Test with Anesthesia, NIBUT and Meibomian Losses and Secretions

**Figure 1:**
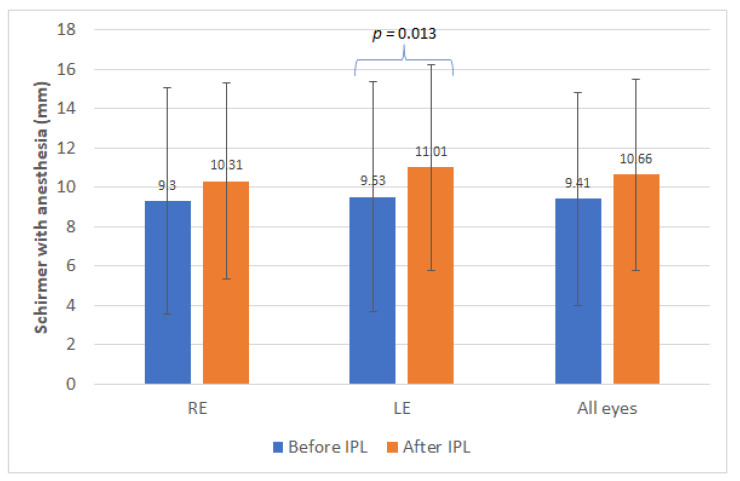
Mean values obtained with the Schirmer test with anesthesia before and after the treatment sessions. Statistical significant differences are displayed with the corresponding *p*-value. IPL: Intense pulsed light. RE, right eye; LE, left eye.

**Figure 2:**
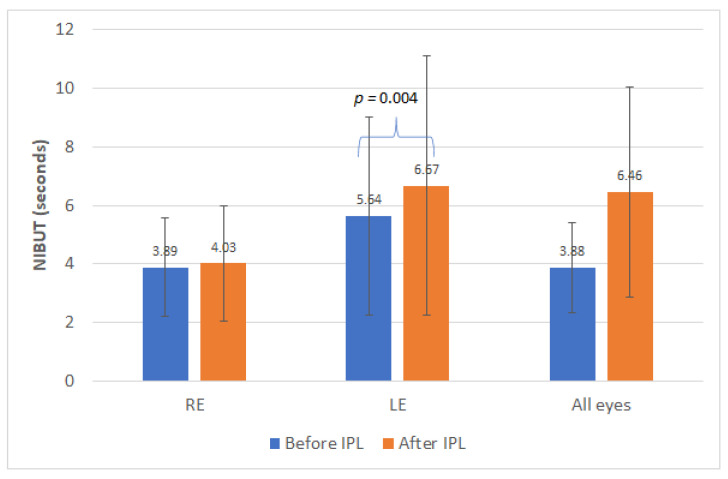
Mean NIBUT values before and after the treatment sessions. Statistical significant differences are displayed with the corresponding *p*-value. NIBUT, non-invasive break-up time.

Table 1 summarizes the main clinical outcomes obtained in this clinical study in the different variables evaluated, considering all eyes (whole sample) and analyzing separately as well the data from right and left eyes. As shown, with the IPL treatment, only statistically significant improvements were found in the outcome of the Schirmer test with anesthesia measured in the left eye (LE) (*p* = 0.013, Wilcoxon test) (Figure 1). Concerning NIBUT measures, only a statistically significant increase in the value of the left eye was detected (*p* = 0.004, Wilcoxon tests) (Figure 2). Likewise, significant changes were found in the distribution of the results of the meibum quality (*p* < 0.001, chi-square test), with more eyes showing clear or yellow secretions after therapy. 

### 3.3. Changes in Tear Film Osmolarity

**Figure 3:**
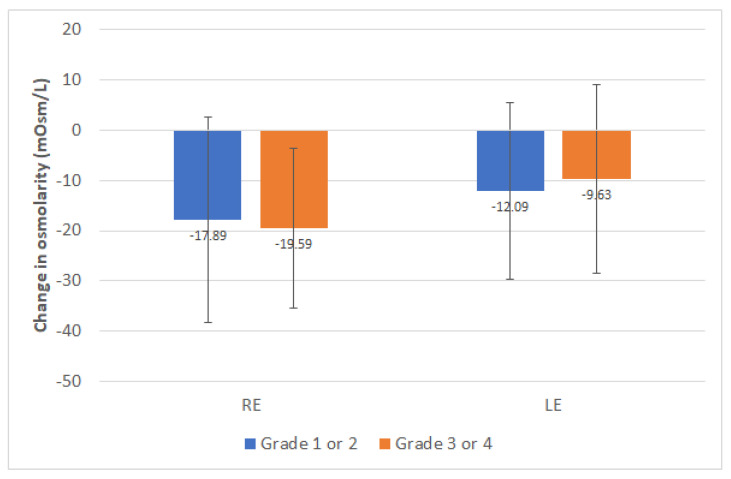
Mean change in tear osmolarity measured in right and left eyes of patients enrolled in the study as a function of the level of the Meibomian gland loss at baseline. Statistical significant differences are displayed with the corresponding *p*-value.

Significant changes were detected in tear film osmolarity in right eye (RE) (*p* < 0.001, Wilcoxon test) and LE (*p* < 0.001, Wilcoxon test), as well as when analyzing the whole sample of eyes (*p* < 0.001, Wilcoxon test) (Table 1). The mean change in tear osmolarity in RE and LE was −19.57 mOsm/L (median: −15.00, range: −110.00 to 25.00 mOsm/L) and −10.71 mOsm/L (median: −11.00; range: −47.00 to 41.00 mOsm/L), respectively. The change is osmolarity in RE correlated significantly with the baseline osmolarity (r = −0.636, *p* < 0.001, Spearman), as well as the change in osmolarity in LE with the osmolarity value before treatment (r = −0.620, *p* < 0.001, Spearman). In addition, there was a significant correlation of the change in osmolarity in the whole sample of eyes with the baseline osmolarity value (r = −0.585, *p* < 0.001, Spearman). This means that more reduction of osmolarity was present in those eyes with higher values of osmolarity at baseline. No significant differences in the reduction in osmolarity between those eyes with a baseline meibomian gland loss grade 1 or 2 and those with grade 3 or 4 were found (right eye, *p* = 0.565; left eye, *p* = 0.404) (Figure 3). Finally, a trend to more reduction of osmolarity in the whole sample of eyes was found in those cases with granular or solid meibomian secretions at baseline compared to those with clear or yellow secretions, but the difference did not reach statistical significance (−12.36 ± 14.43 vs. −20.56 ± 17.46, *p* = 0.063).

### 3.4. Changes in Symptomatology

The change in OSDI with the therapy ranged from −27.00 to 11.00, with a mean and median values of −8.61 and −8.50, respectively (*p* < 0.001, paired Student *t* test). This change was found to be significantly correlated with the baseline value of OSDI (r = −0.489, *p* < 0.001, Pearson) (more reduction in OSDI score with IPL in those cases with worse baseline OSDI), with no significant correlations with the rest of baseline parameters evaluated (−0.282 ≤ r ≤ 0.046, *p* ≥ 0.054, Spearman).

### 3.5. Predictive Model for the Change in Symptomatology with IPL Therapy

Change in OSDI with therapy was found to be significantly related to some baseline data obtained according to the following expression (*p* = 0.001; R^2^: 0.325, adjusted R^2^: 0.276; Durbin-Watson: 1.749):

Change OSDI = 10.99 − 0.35 × OSDI_baseline_ − 1.03 × NIBUT_RE-LE_ − 2.03 × MQ_baseline_ (1)

where OSDI, ocular surface disease index; NIBUT, mean value of the break-up time of right and left eye; MQ, meibum quality (0, clear liquid; 1, yellow liquid; 2, granular; 3, solid).

The homoscedasticity of this model was confirmed by the normality of the unstandardized residual distribution (*p* = 0.893) and the absence of influential points or outliers (mean Cook’s distance = 0.024 ± 0.029).

### 3.6. Comparison between Evaporative and Mixed Dry Eye

In the sample evaluated, a total of 110 patients (56.4%) had an evaporative dry eye, whereas a total of 85 patients (43.6%) showed a mixed dry eye. A comparative analysis of the change achieved in both types of dry eye has been also performed. No statistically significant differences were found in terms of change in OSDI (*p* = 0.209, Mann-Whitney test), tear osmolarity (RE, *p* = 0.526; LE, *p* = 0.120, Mann-Whitney test) and NIBUT (RE, *p* = 0.900; LE, *p* = 0.522, Mann-Whitney test) after therapy in both types of dry eye. Likewise, no statistically significant differences among evaporative and mixed dry eye groups were found in the level of quality of the Meibum after therapy (*p* = 0.390, Chi-square test).

### 3.7. Adverse Events

There were no ocular or facial adverse events or side effects related to the combined light treatment. Only five patients (2.56%) referred no subjective improvement after the treatment. Furthermore, two patients (1.02%) described transient episodes of visual blurring and eye itching after the first session of treatment that resolved spontaneously.

## 4. Discussion

This non-comparative prospective case series has shown, as in previous studies, that IPL-based therapy can be an effective treatment for dry eye with MGD associated [5,6,7,8,9,10,11,12,13,14,15,16,17,18,19,20,21,22]. In agreement with the mechanism of action of the IPL technology, a significant change was observed in the secretion of the meibomian glands, with more eyes showing clear or yellow secretions after therapy. Several authors have also reported significant improvements in meibomian secretion quality after IPL therapy [6,11,12,16]. Gupta et al. [6] reported in a multicenter cohort study involving 100 patients with diagnosis of dry eye and MGD treated with IPL that there was a significant decrease in meibum viscosity scoring (mean: −1.1, range: −3 to 0) and a significant increase in oil flow score (mean: 0.9, range: −0.5 to 2.0). These changes in meibomian secretions are the responsible for the generation of a more consistent lipid layer [5,9,12] leading to increased values of BUT [5,8,9,11,12,13,14,15]. In the current sample, no significant changes were detected in NIBUT. In the current sample, a trend to reduction in the measurement of the NIBUT was also found, but only statistical significance was found in those changes detected in the left eye. It should be considered that the follow-up and method used to measure the NIBUT differ significantly among studies [5,6,8,9,11,12,13,14,15]. Craig et al. [5] found that NIBUT increased significantly from baseline to the end of treatment IPL sessions in a sample of 28 subjects participating in a contralateral study, but the tear evaporation rate did not differ significantly between treated and control eyes at any visit. Ocak and colleagues [13] found that eyes with mild and moderate meibomian gland dropout atrophy did not have an immediate effect on OSDI scores and NIBUT, starting the improvement at 1 month. It should be considered that around half of the sample had a grade 1 or II meibomian gland loss in our series. In any case, the aim of the current study was to define predictors of the improvement of the symptomatology immediately after finishing IPL therapy. Future studies should confirm if these predictive factors are also valid for the results in the long term.

In the current sample, tear osmolarity was significantly reduced in both right and left eyes after IPL therapy, confirming the trend to a recovery of the ocular surface homeostasis. This contrasts with the results reported by Vigo et al. [9] who did not find significant differences in tear osmolarity, but after three IPL sessions. Vergés et al. [15] observed a significant reduction in tear osmolarity between baseline and final visit (316 ± 18 mOsm/L vs. 301 ± 12 mOsm/L, *p* < 0.007) in a sample of MGD-associated dry eyes treated with IPL. This change in tear osmolarity with IPL seems to be the main consequence of the significant change in the lipidic secretions from meibomian glands, allowing a more stable lipid layer and a control of the concentration of electrolytes in the aqueous phase of the tear film. This osmolarity regulation achieved with IPL may be the main causal factor for its great impact on the inflammatory cycle, with significant reduction of inflammatory markers in tears (especially IL-17A and IL-6), as demonstrated in previous studies [7,9]. In the current sample, a significant negative correlation was found between the change in osmolarity induced by IPL and the baseline level of tear osmolarity, indicating that more reduction of this parameter is achieved in those eyes showing a worse baseline osmolarity alteration. This confirms the potential of IPL therapy as a regulator of the tear osmolarity and therefore promoting less inflammatory events. On the other hand, more reduction of mean osmolarity of right and left eyes was found in those cases with granular or solid meibomian secretions at baseline, although the change was only statistically significant for the left eyes. This confirms the therapeutic effect of IPL in MGD. In summary, an altered production and quality of meibomian secretions can be considered as a potential etiological factor of the degradation of the ocular surface homeostasis and the presence of uncontrolled levels of tear osmolarity that can be restored with IPL therapy.

Concerning the results of the Schirmer test with anesthesia, only the change with IPL was statistically significant for the left eyes. This significant change in tear secretion with the Schirmer test was also reported by Mejía et al. [8] in a sample in which most of patients had a mixed dry eye, with reduction of the aqueous component too. Arita et al. [24] evaluated the outcomes of IPL therapy in patients with refractory aqueous-deficient dry eye (contact lens wear, previous ocular surgery, Sjögren syndrome or rheumatoid arthritis) accompanied by mild MGD and found a significant improvement in meibum grade and lid margin abnormality scores, but not in Schirmer I test outcomes. More research in needed to determine the real direct or indirect impact of IPL on aqueous secretion in dry eye patients and which is the exact mechanism of action leading to potential changes in this type of secretion with IPL.

All these clinical changes after IPL were associated to an improvement of symptomatology evaluated by means of the OSDI score, as in previous studies using IPL for the treatment of dry eye with MGD [6,10,11,12,13,14,15,16]. This change in OSDI with IPL was significantly correlated with the level of baseline OSDI, with more potential of improvement in those eyes with severe dry eye-related disturbances. Furthermore, to our knowledge, this study is the first in providing a proposal of model of prediction of the change expected in OSDI with IPL as a function of the baseline conditions. This model considered the following variables: baseline OSDI, mean NIBUT of right and left eyes, and the level of meibum quality. Specifically, more improvement in symptomatology measured with the OSDI questionnaire is expected in MGD-associated dry eyes with severe symptomatology, granular or solid meibomian secretions and reduced levels of the mean NIBUT of both eyes. Future studies should be conducted to validate and refine this model to confirm the clinical usefulness of the model to define a prognosis of the IPL treatment before initiating it.

Concerning the limitations of the study, it should be acknowledged that it is a non-comparative case series and the results obtained have not been compared to those obtained in a control group. In any case, the aim of this article was not to evaluate the efficacy of the IPL treatment. The aim was to characterize the change induced by an IPL-based treatment in eyes with dry eye and MGD, defining potential prognostic factors. The combination of IPL and pharmacological treatment in mixed dry eyes can be considered as another limitation of the current study. Specifically, cyclosporine and autologous serum was used in such cases because it was crucial to treat the hyposecretory component of the dry eye as the IPL therapy only has an impact on the evaporative component of the dry eye. However, it is important to mention that both types of dry eye, evaporative and mixed, obtained the same results after therapy, with no significant differences between dry eye groups. This allows to confirm the beneficial effect of this combination of IPL and pharmacological protocol in dry eyes with both evaporative and hyposecretory components, obtaining a similar outcome than eyes with only evaporative component.

## 5. Conclusions

In conclusion, IPL therapy is an effective option to improve symptomatology in dry eyes associated to MGD, with an additional improvement in clinical signs, such as tear film osmolarity. The improvement in symptomatology with this type of treatment can be predicted consistently using a linear equation considering some baseline parameters, such as OSDI, NIBUT and meibum quality grade. This model should be validated in other different samples evaluating the outcomes of IPL.

## Figures and Tables

**Table 1:** Summary of the preoperative and postoperative clinical data obtained in the analyzed sample.

Mean (SD) Median (Range)	Preoperative	Postoperative	*p*-Value (Test)
OSDI	24.59 (9.06)	13.16 (9.96)	<0.001
	23.00 (15.00 to 50.00)	10.00 (0.00 to 40.00)	(Paired Student t)
Schirmer with anesthesia (mm)			
RE	9.30 (5.76)	10.31 (4.99)	0.365
	9.00 (0.00 to 25.00)	10.00 (0.00 to 20.00)	(Wilcoxon)
LE	9.53 (5.84)	11.01 (5.21)	0.013
	9.00 (0.00 to 27.00)	10.00 (0.00 to 20.00)	(Wilcoxon)
*p*-value RE vs. LE	0.464 (Wilcoxon)	0.134 (Wilcoxon)	
All eyes	9.41 (5.44)	10.66 (4.86)	0.058
	9.00 (0.00 to 25.00)	10.00 (0.00 to 20.00)	(Paired Student t)
NIBUT (s)			
RE	3.89 (1.70)	5.64 (3.38)	0.110
	3.80 (1.10 to 6.70)	4.95 (1.20 to 16.80)	(Wilcoxon)
LE	4.03 (1.97)	6.67 (4.44)	0.004
	3.70 (1.00 to 6.80)	5.05 (1.00 to 16.80)	(Wilcoxon)
*p*-value RE vs. LE	0.759 (Wilcoxon)	0.071 (Wilcoxon)	
All eyes	3.88 (1.55)	6.46 (3.60)	<0.001
	3.80 (1.00 to 6.80)	5.50 (1.20 to 16.80)	(Wilcoxon)
Tear osmolarity (mOsm/L)			
RE	321.33 (12.30)	303.54 (14.90)	<0.001
	317.00 (310.00 to 370.00)	304.00 (236.00 to 336.00)	(Wilcoxon)
LE	319.27 (12.09)	307.05 (15.25)	<0.001
	315.00 (310.00 to 359.00)	307.00 (278.00 to 375.00)	(Wilcoxon)
*p*-value RE vs. LE	0.033 (Wilcoxon)	0.239 (Wilcoxon)	
	320.29 (10.44)	305.23 (11.72)	<0.001
All eyes	317.75 (310.00 to 353.00)	304.50 (276.00 to 337.00)	(Wilcoxon)
Meibomian gland loss (grade 1/grade 2/grade 3/grade 4)			
RE	0.0%/53.4%/44.2%/2.4%	6.5%/67.7%/25.8%/0.0%	0.661 (Chi-square)
LE	0.0%/48.5%/47.9%/3.6%	10.8%/63.4%/24.7%/1.1%	0.166 (Chi-square)
All eyes	0.0%/50.9%/46.1%/3.0%	8.6%/65.6%/25.3%/0.5%	0.077 (Chi-square)
Expression of Meibomian glands (clear/yellow/granular/solid)	15.8%/30.9%/27.9%/25.5%	22.8%/52.6%/22.8%/1.8%	<0.001 (Chi-square)

Abbreviations: OSDI, ocular surface disease index; RE, right eye; LE, left eye; NIBUT, non-invasive break-up time.

## Data Availability

Data available on request due to privacy/ethical restrictions.
